# Diagnostic performance evaluation of hepatitis B e antigen rapid diagnostic tests in Malawi

**DOI:** 10.1186/s12879-021-06134-3

**Published:** 2021-05-27

**Authors:** Alexander J. Stockdale, Niza M. Silungwe, Isaac Thom Shawa, Benno Kreuels, Melita A. Gordon, Anna Maria Geretti

**Affiliations:** 1 Malawi-Liverpool-Wellcome Programme, Blantyre, Malawi; 2 Institute of Infection, Veterinary and Ecological Sciences, University of Liverpool, Ronald Ross Building, 8 West Derby Street, Liverpool, L69 7BE UK; 3 University of Malawi College of Medicine, Blantyre, Malawi; 4 Department of Tropical Medicine, Bernhard Nocht Institute for Tropical Medicine, Hamburg, Germany; 5 First Department of Medicine, University Medical Centre, Hamburg-Eppendorf, Hamburg, Germany

**Keywords:** Hepatitis B, Hepatitis B e antigens, Reagent kits, diagnostic, Sensitivity and specificity, Malawi, Africa south of the Sahara

## Abstract

**Background:**

The World Health Organization (WHO) has targeted a reduction in viral hepatitis-related mortality by 65% and incidence by 90% by 2030, necessitating enhanced hepatitis B treatment and prevention programmes in low- and middle-income countries. Hepatitis B e antigen (HBeAg) status is used in the assessment of eligibility for antiviral treatment and for prevention of mother-to-child transmission (PMTCT). Accordingly, the WHO has classified HBeAg rapid diagnostic tests (RDTs) as essential medical devices.

**Methods:**

We assessed the performance characteristics of three commercially available HBeAg RDTs (SD Bioline, Alere, South Africa; Creative Diagnostics, USA; and Biopanda Reagents, UK) in two hepatitis B surface antigen-positive cohorts in Blantyre, Malawi: participants of a community study (*n* = 100) and hospitalised patients with cirrhosis or hepatocellular carcinoma (*n* = 94). Two investigators, blinded to the reference test result, independently assessed each assay. We used an enzyme-linked immunoassay (Monolisa HBeAg, Bio-Rad, France) as a reference test and quantified HBeAg concentration using dilutions of the WHO HBeAg standard. We related the findings to HBV DNA levels, and evaluated treatment eligibility using the TREAT-B score.

**Results:**

Among 194 HBsAg positive patients, median age was 37 years, 42% were femaleand 26% were HIV co-infected. HBeAg prevalence was 47/194 (24%). The three RDTs showed diagnostic sensitivity of 28% (95% CI 16–43), 53% (38–68) and 72% (57–84) and specificity of 96–100% for detection of HBeAg. Overall inter-rater agreement κ statistic was high at 0.9–1.0. Sensitivity for identifying patients at the threshold where antiviral treatment is recommended for PMTCT, with HBV DNA > 200,000 IU/ml (39/194; 20%), was 22, 49 and 54% respectively. Using the RDTs in place of the reference HBeAg assay resulted in 3/43 (9%), 5/43 (12%) and 8/43 (19%) of patients meeting the TREAT-B treatment criteria being misclassified as ineligible for treatment. A relationship between HBeAg concentration and HBeAg detection by RDT was observed. A minimum HBeAg concentration of 2.2–3.1 log_10_IU/ml was required to yield a reactive RDT.

**Conclusions:**

Commercially available HBeAg RDTs lack sufficient sensitivity to accurately classify hepatitis B patients in Malawi. This has implications for hepatitis B public health programs in sub-Saharan Africa. Alternative diagnostic assays are recommended.

**Supplementary Information:**

The online version contains supplementary material available at 10.1186/s12879-021-06134-3.

## Background

In sub-Saharan Africa, chronic infection with hepatitis B virus (HBV) is the principal cause of liver cirrhosis and hepatocellular carcinoma (HCC) [1]. The World Health Assembly recently adopted targets to tackle viral hepatitis including a 65% reduction in mortality by 2030 [2]. Meeting this goal will necessitate an ambitious increase in HBV screening and treatment activities. Without implementation of adult HBV treatment programmes, it is projected that deaths from chronic HBV will continue to rise beyond 2030 [3]. Accordingly, antiviral treatment programmes are currently being introduced across several sub-Saharan African countries [4, 5].

Ascertainment of hepatitis B e antigen (HBeAg) status is fundamental to the clinical classification of chronic HBV. Guidelines from the World Health Organization (WHO) and hepatology associations recommend use of HBeAg as a component an assessment of eligibility for antiviral treatment [6–9]. Determination of HBeAg status is also central to a proposed simplified treatment protocol for Africa, which uses HBeAg status and ALT concentration [10]. In settings where HBV DNA quantification is unavailable, HBeAg may also be used to select which pregnant patients will require antiviral therapy to prevent mother-to-child transmission (PMTCT), as a surrogate marker of high HBV DNA concentration, and has been recommended in recent WHO guidelines, although it is an imperfect correlate [11–13].

In low- and middle-income countries, there are several potential barriers to accessing enzyme immunoassay (EIA), chemiluminescence immunoassays (CLIA) or electrochemiluminescence assays (ECA) for HBeAg detection. These include cost, limited laboratory capacity, the need for a cold supply chain for reagents, a reliable electricity supply and the lack of laboratory equipment in decentralised rural settings where the majority of the population lives [14]. In many hospitals in sub-Saharan Africa, immunochromographic rapid diagnostic tests (RDTs) are routinely and widely used for diagnosis of infectious diseases, both at the point of care and in hospital laboratories. They offer many potential advantages including room temperature transport and storage, no requirement for an electricity supply (other than for assays using plasma or serum requiring centrifugation), minimal training requirements, rapid time to a result (usually within 15 min), and healthcare worker familiarity with RDT devices [15]. For assessing eligibility of antiviral treatment and for PMTCT, WHO recently listed HBeAg RDTs as a essential diagnostic tests [16].

A report from Senegal in West Africa showed low sensitivity of three commercially available HBeAg RDTs relative to the Architect chemiluminescence testing platform [17]. Further diagnostic performance evaluation studies in sub-Saharan Africa are warranted since this finding might have significant implications for the design and implementation of HBV treatment and prevention activities.

We aimed to assess the diagnostic performance characteristics of HBeAg RDTs in chronic HBV patients in two hepatitis B surface antigen (HBsAg) positive cohorts in Malawi: a community study in an urban township and in patients with cirrhosis or hepatocellular carcinoma in a tertiary hospital in Blantyre, using a commercial plate enzyme-linked immunosorbent assay (ELISA) as a reference test. Samples were tested by two independent operators using three commercially available RDTs. Results were related to HBeAg concentration and also analysed for their ability to correctly identify patients with HBV DNA load > 200,000 IU/ml.

## Methods

### Community study

A serological survey was conducted in Ndirande township from December 2016–April 2018 based on a demographic census as part of the Strategic Typhoid alliance across Africa and Asia (STRATAA) study [18]. Ndirande is a large urban township located in northeast Blantyre. Participants from the census were selected for participation in the serosurvey using single-stage random sampling with replacement for refusals at the household level. If a replacement was not available in the household, we made an additional random selection for replacement from the census population. We tested 6073 randomly selected participants from the serosurvey for hepatitis B surface antigen (HBsAg). We returned to households to invite all HBsAg positive individuals aged over 16 years to participate in a detailed study of HBV disease burden. We additionally invited the household contacts of HBsAg+ individuals for testing; HBsAg+ household contacts were included in this analysis. Samples were collected in community centres close to participants homes and following collection, were stored in temperature monitored cool boxes between 2 and 8 °C prior to transportation to the study laboratory.

### Hospital study

For the hospital study, we prospectively recruited consecutive patients from a tertiary referral hospital (Queen Elizabeth Central Hospital) in Blantyre who had cirrhosis or hepatocellular carcinoma (HCC). Between November 2017 and April 2019, research nurses screened all patients on weekdays in medical and surgical wards, the endoscopy unit and medical clinics using screening criteria to elicit symptoms or signs suggestive of liver disease or HCC. Potentially eligible individuals underwent transient elastography with a study nurse and ultrasound performed by AS. Eligible participants had liver stiffness ≥9.5 kPa after fasting for 3 h (Fibroscan 430 Mini, Echosens, Paris, France) or a hepatic lesion consistent with hepatocellular carcinoma based on ultrasound features using a standardised protocol. We excluded participants with falsely elevated liver stiffness measurements that were unlikely to represent cirrhosis, comprising those with evidence of transaminitis (ALT> 2x upper limit of normal) without ultrasound features of chronic liver disease, patients with right heart failure (IVC dilated > 2.5 cm at level of 1 cm below the cavoatrial junction and/or dilated hepatic veins on ultrasound with clinical evidence of cardiac failure), obstructive jaundice with a dilated biliary system visible on ultrasound and hepatic lesions not consistent ultrasonographically with HCC.

### Laboratory methods

All laboratory work was conducted at the Malawi-Liverpool-Wellcome Trust Clinical Research Laboratories in Blantyre, Malawi. All kits and reagents, including those of the reference ELISA test, were shipped to the testing facility using temperature-controlled shipments and stored in a temperature-monitored cold room at 4 °C until use, within the manufacturer specified expiry date of the kits. Following collection, participant serum tubes (Vacutainer SST II, BD, Franklin Lakes, New Jersey, USA) were centrifuged at 1500×g for 10 min, and dipotassium ethylenediaminetetraacetic acid (K2 EDTA, BD) plasma tubes at 1200×g for 10 min, separated and stored at − 80. Participant sera were tested for HBsAg using the Monolisa HBsAg ULTRA commercial plate ELISA (Bio-Rad, Marnes-la-Coquette, France) in accordance with the manufacturer’s instructions. All HBsAg positive results were confirmed by retesting in duplicate.

HBV DNA was quantified using an in-house quantitative real time PCR calibrated with the 4th International WHO standard. DNA was extracted from 200 μl of plasma using Qiamp DNA mini (Qiagen, Hilden, North Rhine-Westphalia, Germany) and eluted into 60 μl of Tris-EDTA buffer with carrier RNA added at a concentration of 10 ng/μl. A pipetting robot (Qiagility, Qiagen) was used to transfer 15 μl of extracted DNA template, primers (400 nM) and probe (200 nM) and 25 μl of mastermix (Taqman Universal Mastermix, Applied Biosystems, Foster City, CA, USA) onto a 96 well 0.2 ml PCR plate. Primers and probes were as previously published [19] with a FAM reporter dye and a ZEN/IowaBlack FQ quencher probe (Integrated DNA Technologies, Leuven, Belgium). Real time PCR was conducted using the following PCR conditions: 95 °C for 10 min, then 42 cycles of 95 °C for 15 s and 60 °C for 1 min with ROX background adjustment using a Quantstudio Flex 7 (Thermofisher Scientific, Waltham, MA, USA). The lower limit of quantification (LLQ) of the assay was determined as 34 IU/ml and the assay linear range was 1.58 log to 8.58 log_10_ IU/ml. All HBsAg positive samples were tested for HBeAg using the Monolisa HBeAg plus commercial plate ELISA (Bio-Rad). The assay has an analytical sensitivity of 0.64 IU/ml [0.49–0.84] according to manufacturer data, assessed by dilution of the Paul-Ehrlich Institute reference standard. Sera were initially tested qualitatively for HBeAg in accordance with manufacturer instructions. To quantify HBeAg concentration, the 1st WHO International Standard for HBeAg (Paul-Ehrlich Institute, Langen, Germany) was serially diluted 1:2 (from 100 to 0.4 IU/ml) using human serum negative for HBsAg and HBeAg, and tested alongside patient samples using Monolisa HBeAg plus (Bio-Rad). Samples exceeding the range of the standard were diluted and repeated until within range of the standard dilutions. Known concentrations of serial dilutions of the WHO standard were plotted against absorbance at 450/620 nm and a five parameter logistic (5PL) regression curve was fitted to the data [20]. Absorbance at 450/620 nm of HBeAg-positive patients samples plotted on the 5PL standard regression curve was used to quantify HBeAg concentration.

Three commercial RDTs for HBeAg were assessed: i) SD BIOLINE HBeAg (Product code 01FK30, Alere, Kempton Park, Gauteng, South Africa), ii) HBeAg Serum Rapid Test (Cassette), (Catalogue DTS382, Creative Diagnostics, Shirley, NY, USA) and iii) HBeAg Rapid Test (Catalogue RAPG-HBeAg-001, Biopanda Reagents, Belfast, United Kingdom). Participant sera was restored to room temperature prior to testing by two investigators (AS and NS) using each of the three RDTs in accordance with the manufacturers’ instructions. Investigators were blinded to the results of the reference test. The results of each test were recorded by each investigator independently (blinded to the other investigator’s scoring), after which any discordant results were resolved by discussion to achieve consensus.

### Sample size calculation

To evaluate diagnostic test performance with anticipated sensitivity and specificity of 95%, as reported by test manufacturers, with precision of 10%, and based on an anticipated cohort HBeAg prevalence of 20%, we estimated a sample size requirement of 91 participants [21].

### Statistical analysis

Performance characteristics were calculated using exact binomial confidence intervals. Inter-observer agreement was calculated using the Cohen’s kappa [22]. The association between HBeAg concentration and HBV DNA was assessed using Spearman’s ρ statistic. Analyses were performed using Assay Fit Pro (AssayCloud, Nijmegen, Gelderland, The Netherlands). The packages *diagt* [23] and *kappaetc* in Stata v16.1 (Statacorp, College Station, TX, USA) were used to calculate sensitivity, specificity, positive and negative predictive values and diagnostic accuracy.

The study was conducted in accordance with the Standards for Reporting Diagnostic Accuracy Studies 2015 and QUADAS-2 criteria for diagnostic accuracy studies [24, 25].

## Results

### Description of study participants

**Fig. 1:**
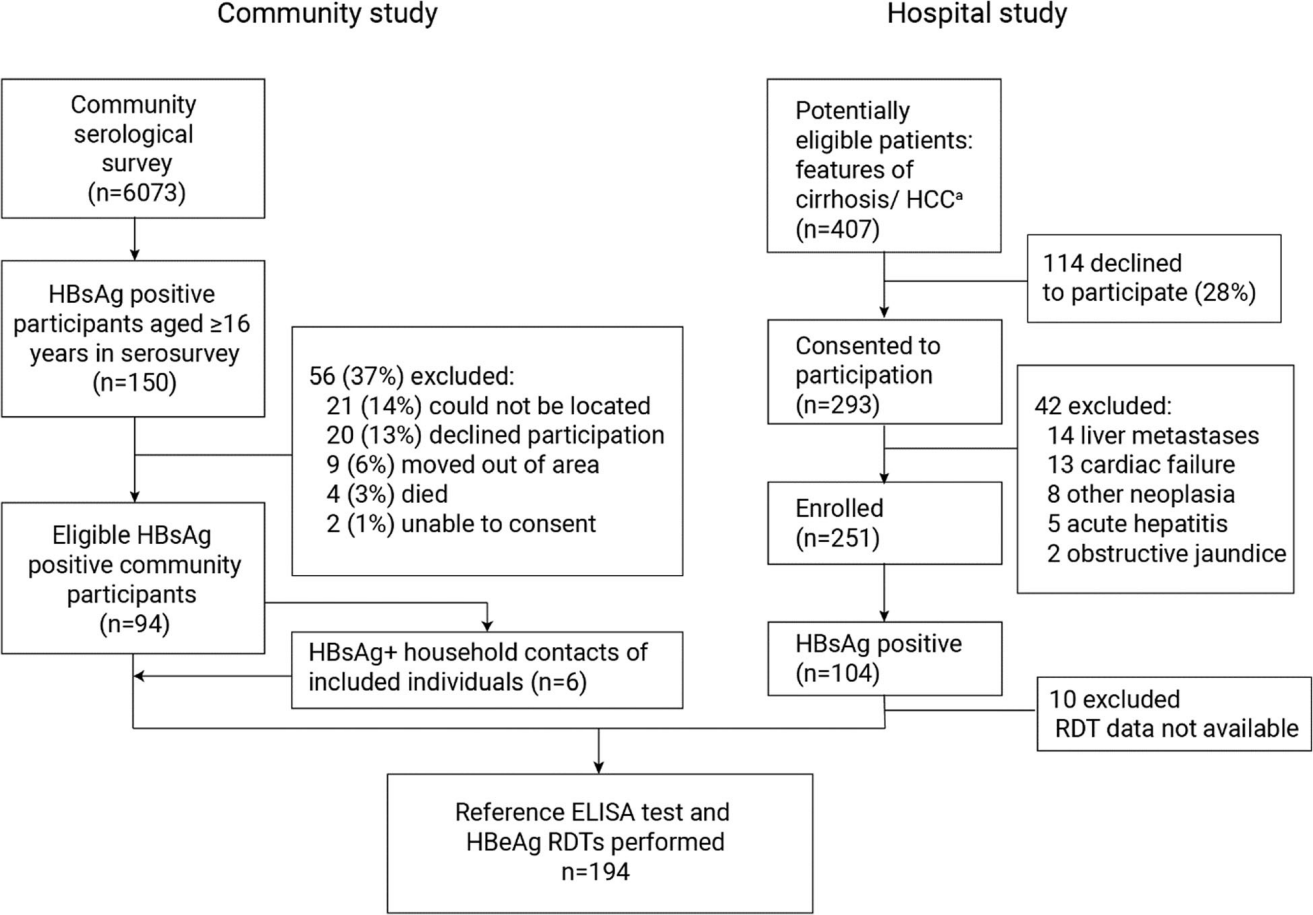
Study recruitment flowchart. ^a^Potentially eligible patients in the hospital study had fasting transient elastography > 9.5 kPa or a hepatic mass

We evaluated 194 HBsAg positive participants, comprising 100 individuals who tested HBsAg positive within a community serosurvey study and 94 hospitalised patients with cirrhosis or HCC (Fig. 1 for study recruitment flowchart). Among the community and hospital populations 24 (24%) and 27 (29%) were HIV positive, respectively. Characteristics of study participants are shown in Table 1. The hospitalised population comprised predominantly men (64/94, 68.1%) and showed a HBV DNA concentration of median 4.2 log_10_ IU/ml, and a median of 6.2 log10 IU/ml among HBeAg positive individuals. Conversely the community population comprised men and women in equal proportions and had a HBV DNA load of median 1.9 log_10_ IU/ml and a liver stiffness of median 4.9 kPa. By qualitative ELISA, 11/100 (11%) of community participants and 36/94 (39%) of hospitalised patients were HBeAg positive.

**Table 1:** Characteristics of HBsAg positive study participants

Characteristic (median (IQR) or n (%))	Population	
	Community	Inpatient
Total number	100	94
Age (years)	36 (29, 41)	40 (34, 45)
Female, n (%)	52 (52.0)	30 (31.9)
HBeAg positive, n(%)^a^	11 (11.0)	36 (38.3)
HBeAg concentration (log_10_ IU/ml)^b^	1.4 (2.1, 2.8)	0.8 (1.4, 2.9)
HBV DNA concentration (log_10_ IU/mL)	1.9 (1.2, 3.1)	4.2 (2.4, 6.3)
HBV DNA > 200,000 IU/ml, n(%)	9 (9.0)	31 (33.0)
HBV DNA > 20,000 IU/ml, n(%)	13 (13.0)	46 (48.9)
HIV status, n (%)		
Positive	24 (24.0)	27 (28.7)
Negative	75 (75.0)	66 (70.0)
Unknown	1 (1.0)	1 (1.1)
CD4 count (cells/mm^3^)^c^	519 (412, 577)	247 (149, 346)
On ART^c^	16 (66.7)	12 (44.4)

Abbreviations: *IQR* Interquartile range, *ART* antiretroviral therapy, *CD4* cluster of differentiation 4, *HBV* hepatitis B virus

^a^By qualitative ELISA reference test

^b^Among HBeAg positive individuals

^c^CD4 count and ART status are described among HIV-positive participants

### Diagnostic performance of HBeAg RDTs

Characteristics of the RDTs and reference ELISA used for HBeAg testing are shown in Table 2. HBsAg Good inter-observer agreement was observed for all RDTs with Cohen’s κ statistic ranging from 0.71 to 1.0. Discrepancies occurred more commonly with the Biopanda Reagents HBeAg rapid test with disagreement on 6/194 tests (3%), relative to 2/194 disagreement (1%) with Creative Diagnostics and no discordance with SD Bioline. In all cases, disagreement was due to faint result bands.

**Table 2:** Comparison of characteristics of HBeAg rapid diagnostic tests and the reference ELISA test

Characteristic	RDT			Reference test
	SD BIOLINE HBeAg (Alere)	HBeAg serum rapid test (Creative Diagnostics)	HBeAg Rapid Test (Biopanda Reagents)	Monolisa HBeAg Plus (Bio-Rad)
Assay format	IC	IC	IC	ELISA
Analyte	Serum or plasma	Serum or plasma	Serum or plasma	Serum or plasma
Sample volume	100ul	120 μl	3 drops (approx. 75 μl)	100 μl
Regulatory approval	None	None	CE marked	CE marked
Wait time	5–20 min	10–20 min	15 min	1.5 h set up, 4 h incubation
Kit storage conditions	2–30 °C	2–30 °C	2–30 °C	2–8 °C
Cost per unit^a^	2.1	4.5	0.4	3.1
Reported sensitivity^b^ (%, (95% CI))	95.5 (88.9–98.2)	96.3 (92.1–98.6)	99.9 (97.7–100)	99.5 (97.3, 100)
Reported specificity^b^ (%, (95% CI))	98.6 (96.1–99.5)	97.9 (96.1–99.1)	98.8 (97.0–99.7)	100 (98.5–100)

Abbreviations: *IC* Immunochromographic, *ELISA* Enzyme linked immunosorbent assay, *CE* Conformité Européenne, *USD* United States Dollars

^a^Price per test in 2018 United States Dollars, excluding shipping

^b^According to manufacturers’ data

**Fig. 2:**
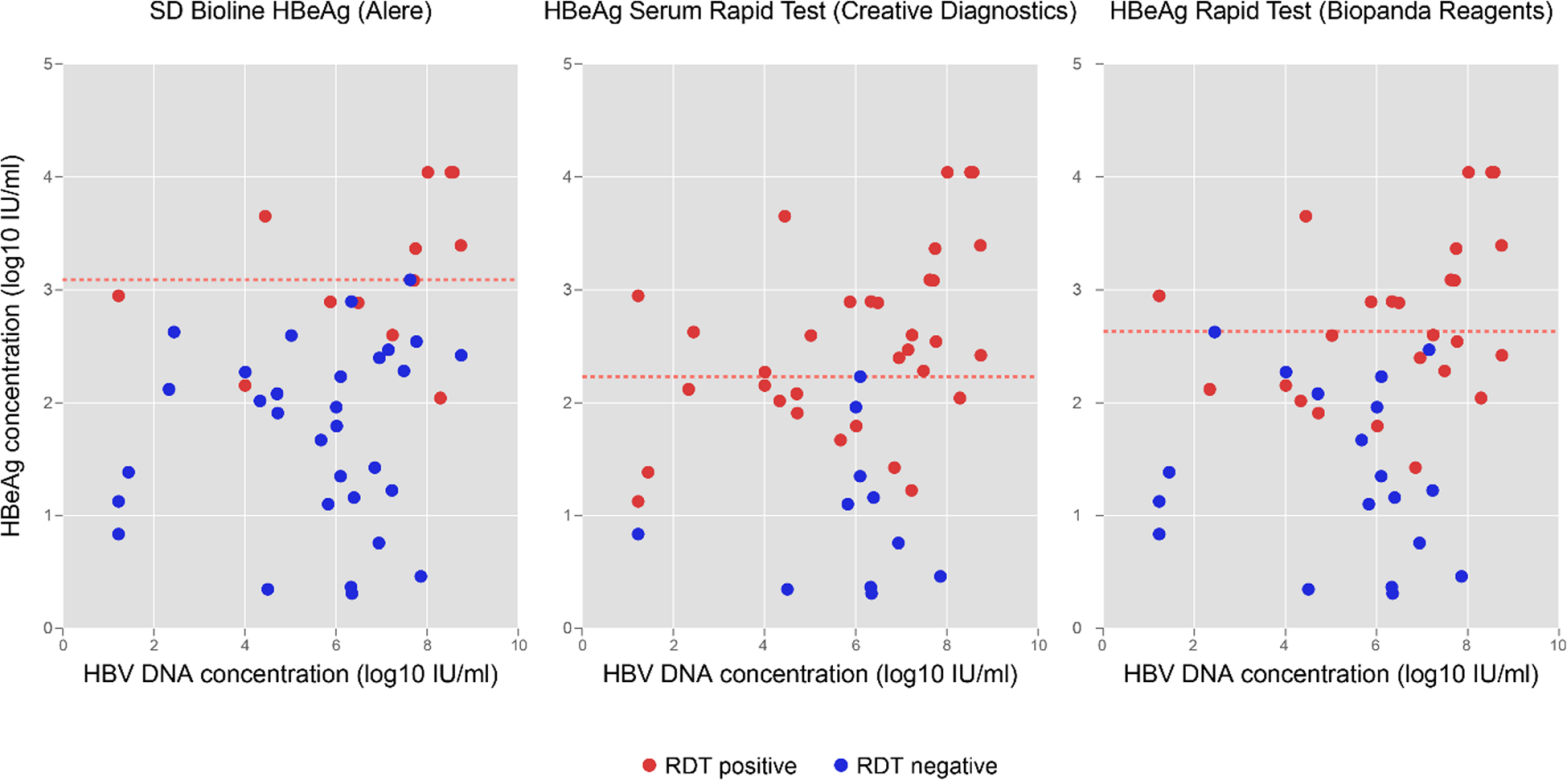
Detection of HBeAg with RDTs relative to HBeAg and HBV DNA concentrations among HBeAg positive samples †. † Red dotted lines refer to threshold HBeAg concentration above which all samples were RDT positive for each of the three RDT assays evaluated. The colour of each point demonstrates the result of HBeAg RDTs, per the figure legend, where red indicates a positive, and blue a negative RDT result. Data shown for 44/47 HBeAg positive individuals; three HIV/HBV HBeAg positive patients on antiretroviral therapy with HBV DNA < 35 IU/ml, are not shown

**Fig. 3:**
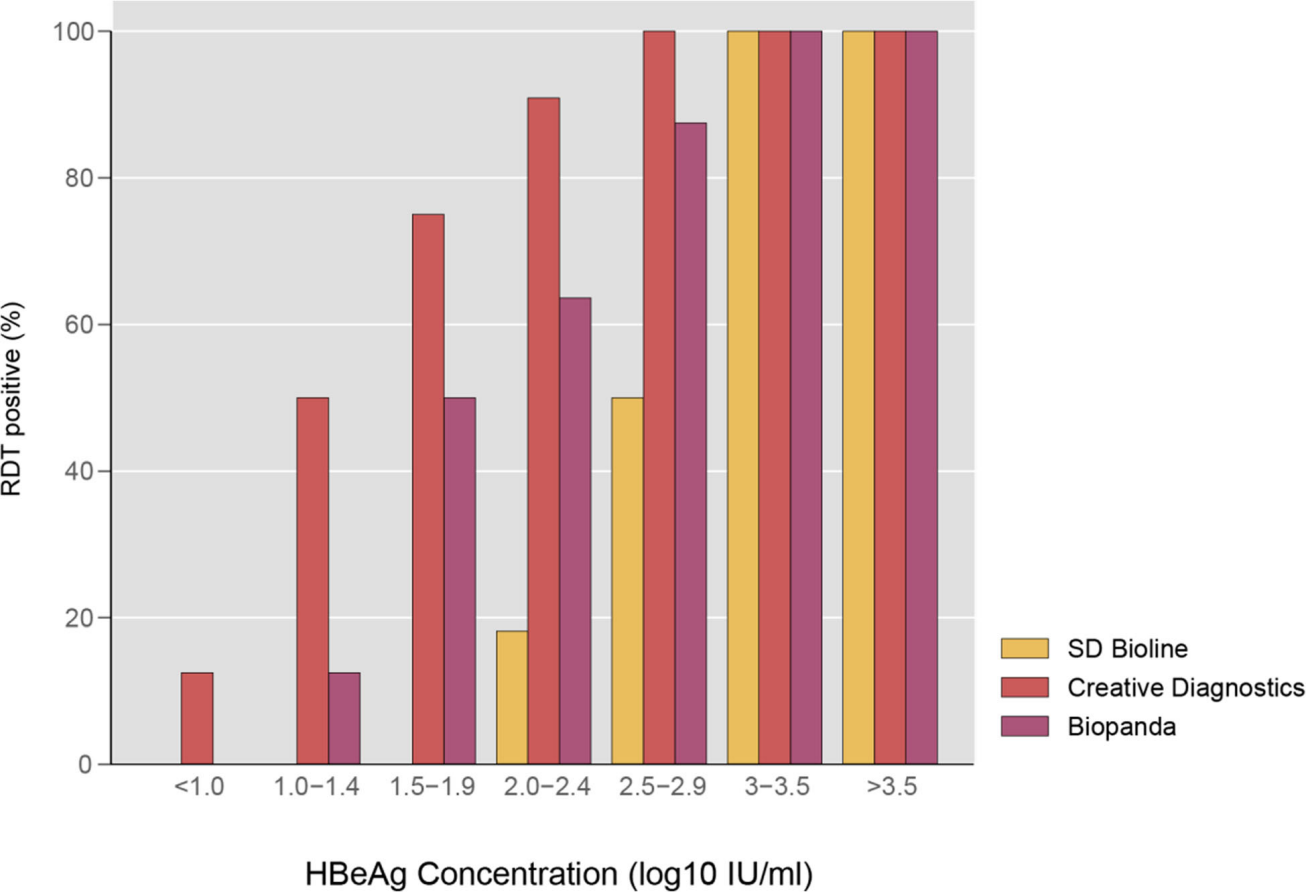
HBeAg rapid diagnostic test results according to HBeAg concentration among HBeAg-positive participants

Diagnostic sensitivity of RDTs relative to the reference plate ELISA was consistently poor on evaluation of all three assays with an overall sensitivity of 28% (95% CI 16–43), 53% (95% CI 38–68) and 72% (95% CI 57–84) for the SD Bioline, Biopanda and Creative Diagnostics tests, respectively (Table 3). The upper bound of 95% confidence intervals for sensitivity for each of the three assays was below 85%. Sensitivity ranged from 36 to 64% in community samples and 25 to 75% in inpatient samples. No association between test sensitivity and HIV status was observed among the three assays (odds ratio 1.3 (95% CI 0.3–5.0, *p* = 0.7), 1.4 (95% CI 0.4–5.8, *p* = 0.6) and 1.1 (95% CI 0.3–3.5, *p* = 0.9), respectively. Overall specificity exceeded > 95% for all assays (Table 3). Cross-tabulated raw data are shown in the Additional file 1. HBeAg detection rate by RDTs was associated with the HBeAg concentration. (Figs. 2 and 3). For the SD Bioline, Creative Diagnostics and Biopanda HBeAg RDTs, the minimum threshold HBeAg concentration yielding consistent HBeAg reactivity was 3.1, 2.2 and 2.6 log_10_ IU/ml respectively.

**Table 3:** Performance evaluation of commercial HBeAg RDTs using a plate ELISA as a reference test

	Diagnostic performance characteristics (95% confidence interval)		
	SD BIOLINE HBeAg (Alere)	HBeAg serum rapid test (Creative Diagnostics)	HBeAg Rapid Test (Biopanda Reagents)
**Overall (*n*** **= 194)**			
κ statistic^a^	1.0 (1.0–1.0)	0.96 (0.92–1.0)	0.89 (0.80–0.98)
Sensitivity (%)	27.7 (15.6–42.6)	72.3 (57.4–84.4)	53.2 (38.1–67.9)
Specificity (%)	100 (97.5–100)	99.3 (96.3–100)	95.9 (91.3–98.5
**Community study (*n*** **= 100)**			
κ statistic ^a^	1.0 (1.0–1.0)	1.0 (1.0–1.0)	0.71 (0.41–1.0)
Sensitivity (%)	36.4 (10.9–69.2)	63.6 (30.8–89.1)	36.4 (10.9–69.2)
Specificity (%)	100 (95.9–100)	100 (95.9–100)	98.9 (93.9–100)
Positive predictive value (%)	100 (39.8–100)	100 (59–100)	80.0 (28.4–99.5)
Negative predictive value (%)	92.7 (85.6–97.0)	95.7 (89.4–98.8)	92.6 (85.4–97.0)
Diagnostic accuracy (%)	93.0 (86.1–97.1)	96.0 (90.1–98.9)	92.0 (84.8–96.5)
**Hospital study (*n*** **= 94)**			
κ statistic^a^	1.0 (1.0–1.0)	0.95 (0.88–1.0)	0.92 (0.83–1.0)
Sensitivity (%)	25.0 (12.1–42.2)	75.0 (57.8–87.9)	58.3 (40.8–74.5)
Specificity (%)	100 (93.8–100)	98.3 (90.8–100)	91.4 (81.0–97.1)
Positive predictive value (%)	100 (66.4–100)	96.4 (81.7–99.9)	80.8 (60.6–93.4)
Negative predictive value (%)	68.2 (57.2–77.9)	86.4 (75.7–93.6)	77.9 (66.2–87.1)
Diagnostic accuracy (%)	71.3 (61.0–80.1)	89.4 (81.3–94.8)	78.7 (69.1–86.5)

Abbreviations: *AROC* Area under the receiver operating curve, *RDT* rapid diagnostic test, *ELISA* enzyme linked immunosorbent assay

^a^Cohens’ κ statistic demonstrates inter-rater agreement

We considered the application of HBeAg as a surrogate for identifying individuals with a HBV DNA > 200,000 (5.3 log_10_) IU/ml, in keeping with the recommended threshold for use of antiviral therapy for prevention of mother to child transmission in WHO guidelines [12]. Among HBeAg positive individuals, a weak correlation was observed between HBeAg concentration and HBV DNA concentration (Spearman’s ρ = 0.35, *p* = 0.02). The sensitivity of HBeAg RDTs for detection of HBV DNA above 200,000 IU/ml for the HBeAg RDTs was 22.2% (95% CI 11.2, 37.1) for SD Bioline, 53.8% (95% CI 37.2, 69.9) for Creative Diagnostics and 48.7% (95% CI 32.4, 65.2) for Biopanda RDTs. By comparison, the sensitivity for the reference test HBeAg ELISA for the detection of HBV DNA > 200,000 IU/ml was 76.9% (95% CI 60.7, 88.9). When this analysis was restricted to the 65 women of childbearing age (aged between 16 and 45 years; median age 34 (IQR 30–38)) included in this study, the sensitivity of HBeAg RDTs for detecting HBV DNA > 200,000 IU/ml was 16.7% (95% CI 0.4–64.1), 50.0% (95% CI 11.8–88.2) and 33.3% (95% CI 4.3–77.7) for SD Bioline, Creative Diagnostics and Biopanda, respectively. The sensitivity of HBeAg RDTs for detecting the treatment threshold of HBV DNA of 20,000 IU/ml as recommended in WHO treatment guidelines, was 19.0% (95% CI 9.9, 31.4) for SD Bioline, 44.8% (95% CI 31.7, 58.5) for Creative Diagnostics and 41.4% (95% CI 28.6, 55.1) for Biopanda RDTs. By comparison, the sensitivity for the reference test HBeAg ELISA for the detection of HBV DNA > 20,000 IU/ml was 62.1% (95% CI 48.4, 74.5).

We next evaluated treatment eligibility using the TREAT-B score, comparing the effect of using RDTs in place of the reference ELISA assay. A total of 43/143 participants (30.1%) met the TREAT-B criteria for starting HBV treatment (with a TREAT-B score ≥ 2), excluding the 51 individuals living with HIV. This comprised 41/67 (61.2%) hospital patients and 2/76 (2.6%) of community patients. Using RDTs instead of the reference assay, a total of 8/43 (18.6%), 5/43 (11.6%) and 3/43 (9.3%) of patients who met TREAT-B treatment criteria, were misclassified as not requiring treatment on the basis of the SD Bioline, BioPanda and Creatine Diagnostics RDTs, respectively. By contrast, 0/101 (0%), 2/101 (1.9%) and 1/101 (1.0%) were classified as requiring treatment based on the SD Bioline, BioPanda and Creatine Diagnostics RDTs, respectively, when evaluated using the reference HBeAg ELISA test, treatment was not required.

## Discussion

We found that commercially available HBeAg RDTs have poor sensitivity in HBsAg positive populations in Malawi when compared to a reference ELISA test. The threshold concentration of HBeAg to allow consistent detection by RDTs ranged from 2.2 to 3.1 log_10_ IU/ml. We found that the assays performed poorly at identification of patients with HBV DNA above 200,000 IU/ml, including among women of childbearing age, or at the 20,000 IU/ml threshold recommended in WHO treatment guidelines and thus may not be suitable to serve as a surrogate marker of high viral load. We further identified that between 9 and 19% of patients meeting the TREAT-B criteria for treatment would be misclassified as ineligible for treatment, by using HBeAg RDTs instead of a HBeAg ELISA test, with a smaller number (between 0 and 2%) misclassified as eligible. Our findings are in keeping with recent evidence from West Africa, with a similar poor sensitivity observed in both studies, potentially suggestive of a generalisable problem with diagnostic sensitivity in patients in sub-Saharan Africa [17].

Our findings have substantial implications for planning and implementation of hepatitis B treatment and prevention activities, and for patient care. The WHO recently added HBeAg RDTs to the list of essential medical diagnostics, and RDTs are often used for HBeAg status ascertainment in resource-limited settings [16]. Furthermore, HBeAg is a central component of a recently proposed treatment eligibility scoring tool for Africa [10] and are included in the WHO guidelines for identifying pregnant women who should receive tenofovir to prevent mother to child transmission of HBV, in settings where HBV DNA quantification is not available [12, 13]. Inadequate clinical sensitivity of HBeAg RDTs may therefore potentially deny patients access to treatment for chronic HBV, and result in missed opportunities to prevent mother-to-child transmission.

HBeAg is an important marker of HBV replicative status and is considered fundamental to classification of disease, according to international guidelines for HBV treatment [6, 7, 9]. HBeAg is a soluble 25 kDa protein encoded from the pre-C transcript of the HBV core open reading frame and acts as an immunomodulatory T cell tolerogen [26, 27]. After translation from pre-genomic RNA, it is post-translationally processed and secreted via the endoplasmic reticulum [27]. Its role in HBV immunology is to attenuate the adaptive response, favouring T-helper 2 over T-helper 1 activation and downregulating toll like receptor expression [26, 28]. Seroclearance of HBeAg and seroconversion to anti-HBe typically signals a reduction of virus replication declining with HBV DNA levels and reduced hepatic inflammation [7]. That HBeAg status is associated with increased disease activity, higher HBV DNA concentration and increased risk of development of liver disease and hepatocellular carcinoma, has led to its widespread use as a biomarker for prognostication and for clinical management [7]. The reduced sensitivity for detection of HBeAg observed in RDTs evaluated in this study is likely to be due to core and pre-core variability relative to strains used to develop these assays. In Malawi genotype A1 predominates and several mutations in the core gene have been described in association with this specific genotype such as G1826T [29, 30].

RDTs have many advantages over more advanced laboratory techniques and are widely used for the detection of many infectious diseases in low and middle income countries [15]. Alternative methods such as ELISA, CLIA and ECA present substantial barriers to routine use in these settings. The HBeAg ELISA reference test we used in this study has an incubation period of 4 h and hands-on laboratory time of 1.5 h, requiring skilled laboratory technicians and batching of samples, resulting in significant delays to returning results to clinicians. Diagnostic platforms based on CLIA or ECA are expensive, require a stable electricity supply and are seldom available outside of central hospitals in many low and middle-income countries. An alternative approach is to quantify HBV DNA which has become more feasible with the advent of point of care cartridge based systems such as the recently developed HBV DNA viral load GeneXpert [1]. HBV treatment programmes in sub-Saharan Africa may share the infrastructure developed for HIV care including molecular platforms currently used for HIV-1 RNA quantification which may also often be used for HBV DNA quantification [14]. This includes use of dried blood spot sampling to facilitate sample storage and transport [31].

This study has significant strengths including an evaluation of an epidemiologically representative community study and of a prospective consecutively recruited hospital with significant liver disease. Both cohorts thus have evaluated these assays in well defined settings where these tools are likely to be used. We performed this evaluation in accordance with international criteria for evalution of a diagnostic assay, including fulfilling reporting criteria and using independent assessment with investigators blinded to the reference assay. The main limitation of this analysis is the potential lack of generalisability to an antenatal cohort to evaluate the use of HBeAg RDTs according to recent WHO mother to child prevention guidelines. Our subgroup analysis among women of childbearing age showed a poor sensitivity for identifying patients with HBV DNA > 200,000 IU/ml, a finding that was consistent with the overall cohort. However, the age structure of this subgroup analysis (median age of 34 years) is notably older than the overall antenatal population of Malawi, where age at first pregnancy is median 19 years, median birth interval is 41 months, and total fertility rate is 4.4 [32]. This may have implications for generalisability from our study to an antenatal population, given that the natural history of HBV frequently involves transition toward HBeAg negative status between adolescence and adulthood. We advocate additional evaluation of RDTs is required in an antenatal population before they are used for this purpose.

## Conclusions

We observed that commercially available HBeAg rapid diagnostic tests have inadequate sensitivity for use in treatment or prevention programmes in Malawi, a finding in keeping with previous data from Senegal. Our findings highlight the importance of ensuring that diagnostic tests undergo evaluation in the environment where they will be used, to reflect local epidemiology, population and viral genetic characteristics. Indeed, performance evaluation data from the manufacturer suggested sensitivity in excess of 95% for each of the evaluated assays. There is a pressing need to develop HBeAg RDTs with improved sensitivity adapted for use in sub-Saharan Africa and validated with locally prevalent HBV genotypes, to facilitate effective HBV treatment and prevention programmes. We recommend that in the interim, alternative HBeAg detection methods or HBV DNA quantification are used instead.

## Supplementary Information

**Additional file 1.** Cross tabulation of hepatitis B e antigen rapid diagnostic tests and ELISA reference test.

## Data Availability

HBeAg evaluation data are presented in full in the Additional file 1. Deindentified individual participant data are available from the corresponding author on reasonable request.

## Abbreviations

CI: Confidence intervals
CLIA: Chemiluminescence immunoassay
DNA: Deoxyribonucleic acid
ECA: Electrochemiluminescence assay
EIA: Enzyme immunoassay
ELISA: Enzyme linked immunosorbent assay
HBeAg: Hepatitis B e antigen
HBV: Hepatitis B virus
HIV: Human immunodeficiency virus
RDT: Rapid diagnostic test
RNA: Ribonucleic acid
WHO: World Health Organisation
