# Supplementary material for: Diagnostic performance evaluation of hepatitis B e antigen rapid diagnostic tests in Malawi
Source: BMC Infect Dis. 2021 May 27;21:487. doi: 10.1186/s12879-021-06134-3 (PMC8157469; doi:10.1186/s12879-021-06134-3)
Supplement: Supplementary file 1 — Additional file 1. Cross tabulation of hepatitis B e antigen rapid diagnostic tests and ELISA reference test. [file 12879_2021_6134_MOESM1_ESM.docx]

**Supplementary Appendix for:**

**Diagnostic Performance Evaluation of Hepatitis B e Antigen Rapid Diagnostic Tests in Malawi**

**Authors:** Alexander J. Stockdale^1,2^, Niza M. Silungwe^1^, Isaac Thom Shawa^1,3^, Benno Kreuels^3,4,5^, Melita A. Gordon^1,2^, Anna Maria Geretti^2^

**Affiliations:**

^1^Malawi-Liverpool-Wellcome Programme, Blantyre, Malawi;

^2^Institute of Infection, Veterinary and Ecological Sciences, University of Liverpool, Liverpool, United Kingdom;

^3^University of Malawi College of Medicine, Blantyre, Malawi;

^4^ Department of Tropical Medicine, Bernhard Nocht Institute for Tropical Medicine and

^5^ 1^st^ Department of Medicine, University Medical Centre, Hamburg-Eppendorf, Hamburg, Germany;

**Supplementary appendix: Cross tabulation of hepatitis B e antigen rapid diagnostic tests and ELISA reference test**

| **Evaluated assay** | **Reference test result** | | |
| --- | --- | --- | --- |
| **RDT result: SD Bioline HBeAg (Alere)** | Positive | Negative | Total |
| Positive | 13 | 0 | 13 |
| Negative | 34 | 147 | 181 |
| Total | 47 | 147 | 194 |
| **RDT result: HBeAg serum rapid test (Creative Diagnostics)** |  |  |  |
| Positive | 34 | 1 | 35 |
| Negative | 13 | 146 | 159 |
| Total | 47 | 147 | 194 |
| **RDT result: HBeAg Rapid Test (Biopanda Reagents)** |  |  |  |
| Positive | 25 | 6 | 31 |
| Negative | 22 | 141 | 163 |
| Total | 47 | 147 | 194 |
